# A Brief Review of Poly(Vinyl Chloride) (PVC) Recycling

**DOI:** 10.3390/polym14153035

**Published:** 2022-07-27

**Authors:** Krzysztof Lewandowski, Katarzyna Skórczewska

**Affiliations:** Faculty of Chemical Technology and Engineering, Bydgoszcz University of Science and Technology, Seminaryjna 3, 85-326 Bydgoszcz, Poland

**Keywords:** poly(vinyl chloride), PVC, recycling

## Abstract

Bearing in mind the aspiration of the world economy to create as complete a closed loop of raw materials and energy as possible, it is important to know the individual links in such a system and to systematise the knowledge. Polymer materials, especially poly(vinyl chloride) (PVC), are considered harmful to the environment by a large part of society. The work presents a literature review on mechanical and feedstock recycling. The advantages and disadvantages of various recycling methods and their development perspectives are presented. The general characteristics of PVC are also described. In conclusion, it is stated that there are currently high recycling possibilities for PVC material and that intensive work is underway on the development of feedstock recycling. Based on the literature review, it was found that PVC certainly meets the requirements for materials involved in the circular economy.

## 1. Introduction

Poly(vinyl chloride) is one of the oldest thermoplastic polymers. Since the beginning of industrial PVC synthesis, in the early 1930s, its production volume has been constantly growing [1]. It is currently third in the world in terms of production volume [2].

Sodium chloride (rock salt) is one of the raw materials used in the synthesis of PVC. As a result, only 43% of the polymer mass comes from petrochemical raw materials. The low carbon footprint of the elements made of PVC with a long service life is an additional ecological advantage. For example, the carbon footprint of the manufacturing stage and the entire life cycle of PVC products can be significantly lower compared to other materials, even those generally considered to be environmentally friendly [3,4,5]. In addition, there are prospects for the further reduction of the carbon footprint of PVC through the use of new technologies for the production of vinyl chloride from natural gas [6,7].

The high economic significance of PVC is the outcome not only of its low production costs but is primarily determined by its good properties, the most important of which are high chemical resistance [8] and favourable mechanical properties, as well as resistance to water and weather conditions [9]. Its good adhesive properties enable printing in, e.g., wallpaper, advertisement and floor-panel manufacturing [10]. The high transparency of this polymer means it is used in the manufacturing of foil, blisters or light-transmitting panels. PVC exhibits numerous unique, additional features, such as resistance to biofilm formation [11], high-impact strength, universal flexibility modification, gloss formability and easy binding. It is classified as a self-distinguishing material (LOI of rigid PVC is approximately 44–49%) [12]. Through the possible application of significant amounts of plasticisers, it enables the obtaining of hard and soft variants, which considerably differ in terms of glass transition temperature and flexibility at a specific operating temperature [13].

**Figure 1:**
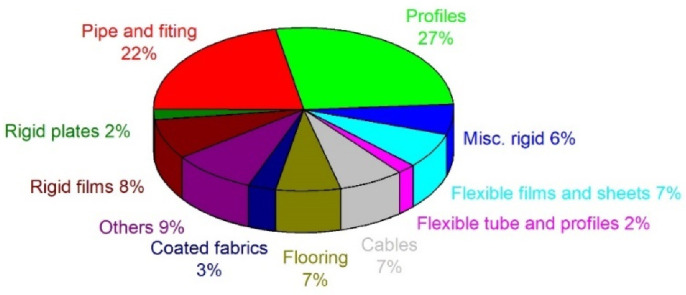
The use of PVC in the European Union, broken down into applications [15].

The use of PVC in the European Union, broken down into applications, is shown in Figure 1. Approximately 70% of PVC output is employed in the construction industry, mainly as window and door profiles, water and sewage pipes, cable insulations, gutters, floor lining, and roof membranes [14].

More than a quarter of polymer products used in medicine is made of PVC, owing to its biocompatibility, chemical stability and resistance to sterilisation. It is used to make flexible blood containers, urine ostomy bags, flexible tubes, inhalation masks, oxygen masks, or PPE such as gloves and footwear [16,17,18]. Moreover, PVC in the form of painting dispersions or mats is used to coat floors and walls, ensuring sanitary safety [19]. PVC is also utilised in the packaging industry as food wrap. Such wrap-foils offer good oxygen barrier properties, translating into a long shelf life of the food [20]. Various blisters for pharmaceuticals [21] and cosmetic packaging are also made of PVC. Plasticised PVC is exploited to manufacture coated fabrics as materials for tarpaulins and coverings for large tents and halls, floor linings and, above all, so-called artificial leather [22], employed in the clothing, automotive and furniture industries [23,24,25]. As far as the automotive industry is concerned, PVC is mainly applied as a material for cable insulation, in addition to the fabrication of fuel hoses and soundproofing mats, as well as for anti-corrosion coatings.

Such widespread and common applications of PVC are correlated with the generation of a waste stream that should be managed in a safe manner.

Poly(vinyl chloride) is mistakenly considered difficult to recycle due to its complex composition and its low thermal stability. This misconception is true not only with respect to public opinion, but also with respect to many people interested in the subject of other polymer materials. However, there are a number of technical possibilities for the management of PVC waste. The aim of this article is to present the possibility of PVC recycling to a wide group of readers, especially those who do not deal with PVC recycling on a daily basis. It is extremely important in the pursuit of a circular economy to be a conscious consumer, processor and scientist.

## 2. PVC Recycling

**Figure 2:**
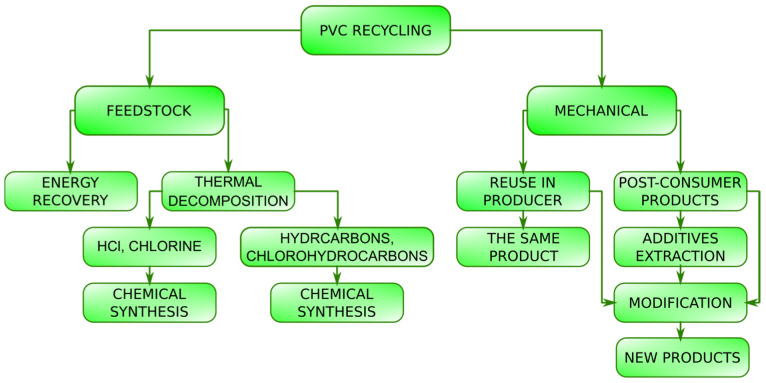
Simplified scheme of poly(vinyl chloride) recycling system.

The basic PVC recycling system is schematically shown in Figure 2. PVC can be subject to both mechanical recycling processes and feedstock recycling.

The most-recommended way to recycle PVC is mechanical recycling. The easiest way is to recycle the material directly in the production plant where the waste is generated. Such waste arises, for example, during the start-up and end of production and the mechanical processing of finished products or waste resulting from production errors. In such a case, with little effort the recycled material can be carefully selected so as not to lead to its contamination. PVC waste after mechanical milling can be used as an admixture for the original material. It is also important that PVC waste processed in the same production plant is of known composition. This allows for its simple modification by adjusting additional PVC components (e.g., process lubricants, thermal stabilisers, increasing the proportion of plasticiser) and designating such material for the production of a different range of products when dosing the original material is impossible.

It is slightly more difficult to obtain the consistency of the composition of the raw material during the recycling of post-consumer materials. In this case, the need to clean the raw material should be taken into account. Additionally, it may be necessary to modify the composition of PVC in order to obtain the specific processing and performance properties required for a new application. In some cases, it may be justified to remove modifiers (e.g., thermal stabilisers or some types of plasticiser); however, this process may turn out to be uneconomical due to the high investment costs related to the purchase of specialised technology.

Another method of PVC-waste management is feedstock recycling. For economic and environmental reasons, this type of recycling should include waste that cannot be mechanically recycled. A relatively simple method of this type of recycling is energy recovery, which consists of gasification of fuels or direct combustion in specialised thermal utilisation plants. Importantly, in the case of energy recovery, PVC can occur as a fraction mixed with other types of waste. However, it should be borne in mind that the resources contained in waste are irretrievably excluded from the circular economy.

A slightly more advanced method of feedstock recycling is the processing of PVC into valuable raw materials for the chemical industry. These processes are carried out in appropriately designed thermal decomposition. In this case, a large investment expenditure related to the construction of specialised installations is required. This type of recycling, in many cases, may turn out to be uneconomical. However, in an attempt to close the circulation of materials in the global economy, such investments may be necessary. It should also be remembered that scientific and technological progress provides new possibilities for processing PVC into other raw materials, as well as prospects for the further development of already existing technologies.

### 2.1. Mechanical Recycling

Poly(vinyl chloride) is considered a polymer material with very limited mechanical recycling. This is due to the misconception of its low thermal stability and dangerous degradation products causing the increased corrosion of processing equipment and the alleged capacity as a threat to people. The proper application of thermal stabilisers allows for the obtaining of PVC material with a very long time of thermal stability, and thus for proper processing [9,26,27].

When considering PVC recycling, we should take into account the fact that, in the processed PVC blends, apart from the aforementioned thermal stabilisers, a number of other additives are used. These are external and internal lubricants, flow modifiers, modifiers of mechanical properties, plasticisers, and, often, a relatively large content of mineral fillers, such as chalk, talc and titanium white. Their use enables the precise adjustment of the processing and functional properties of the processed PVC blend [13,28,29].

In some cases, PVC material recycling can save up to 90% of energy compared to the energy input required for the use of virgin materials. Thus, CO2 emissions are reduced [30].

#### 2.1.1. Post-Production PVC Recycling

Waste with a defined composition, generated mainly in the processing plant, can be directly reprocessed by grinding. It has been proven that unplasticised PVC can be processed several times without clear signs of degradation. In addition, the number of times the same material can be processed can be significantly increased by admixing recyclate with the virgin material in an amount exceeding 30% [31,32].

Ground PVC waste can be directly processed into other products. For technical reasons, it can be processed into granules, although each subsequent processing may reduce the thermal stability of the PVC [31].

PVC waste can be pulverised. In this process, the PVC is crushed to a particle size similar to the original PVC grain. This enables the introduction of PVC recyclate into the virgin PVC at the stage of producing dry blends.

Waste from companies producing windows made of PVC profiles is a relatively large stream of PVC recyclate, with stable properties and compositions of the blend. However, this requires the separation of protective veneers and metal waste from window fittings and plasticised PVC or EPDM, which is used as a material for seals. Complete lines specialised for these purposes are available on the market [33,34,35,36].

**Figure 3:**
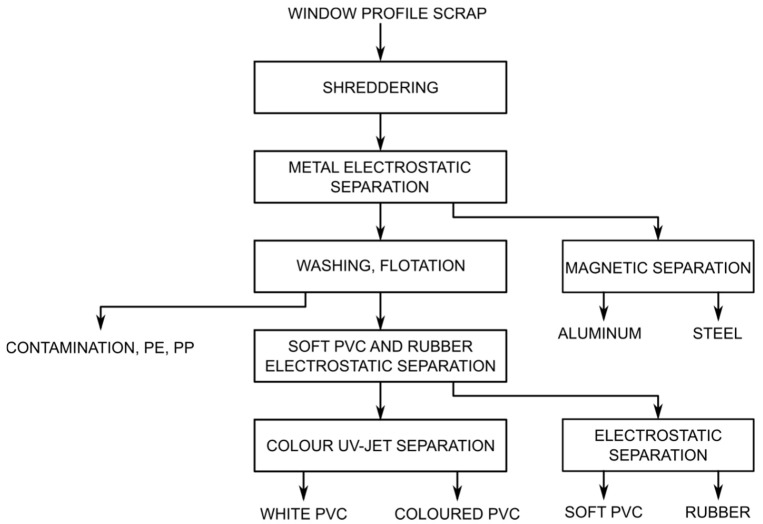
Schematic diagram of the line for recycling PVC window profiles.

Depending on the technology used and the waste material, this process may slightly differ. It can be divided into several successive basic stages, which are presented in the schematic diagram in Figure 3.

In the first stage, a single- or several-stage grinding process takes place, sometimes combined with the separation of the dust fraction. In the next stage, the metal fractions are separated from the polymeric materials by means of electrostatic separators. Metal fractions are subjected to electromagnetic separation in which aluminium is separated from steel. Then, depending on the quality of the material, the polymer fraction is subjected to a washing process in which small amounts of plastics with a density lower than 1 g/cm^3^ are simultaneously separated by means of flotation. They are mainly PP and PE, used as protective veneers for PVC profiles. The cleaned plastic fraction is separated into rubber, soft PVC and unplasticised PVC by means of successive electrostatic separation processes. The white colour fraction is separated from the unplasticised PVC fraction. For this purpose, efficient, modern separators are used, equipped with high-speed cameras that monitor the moving layer of plastic under UV light, and particles of different colours are blown out of the PVC.

PVC recyclates have been successfully used to produce a wide range of composites, often with a high degree of filling. It has been shown that the slight contamination of PVC with incompatible polymers does not significantly affect the properties of these composites [37,38,39].

The mixture of PVC waste with various compositions is also suitable for recycling. It is possible to successfully obtain a material with satisfactory mechanical properties. Additionally, the possibility of PVC modification using a wide range of process grease and fillers allows us to optimise the composition of the blend in terms of the rheological properties. Thus, it is possible to produce multilayer products in the process of co-extrusion. The core of such a profile is made up of a modified recyclate, while the outer layers, which contain virgin PVC, provide specific functional and visual properties, as well as reinforce the recyclate layer with lower mechanical properties [40].

**Figure 4:**
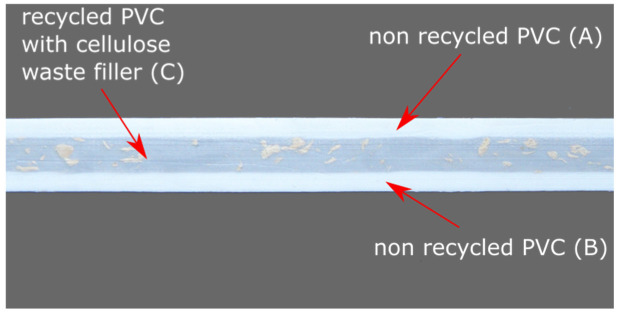
Cross-section of multi-layer material with inner recycled PVC with cellulose filler (C) and non-recycled PVC outside layers (A,B).

**Figure 5:**
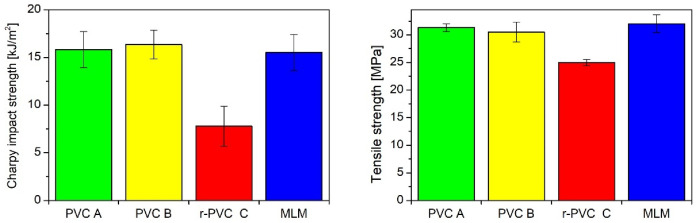
Comparison of the mechanical properties of recycled PVC (C) and non-recycled PVC (A,B) with the multi-layer material made of them (MLM) (errors bars represent standard deviation).

A cross-section of such a material, with a clearly visible internal layer made of recycled material with cellulose filler (C) and external layers made of unrecycled PVC (A, B), is shown in Figure 4. Figure 5 compares the mechanical properties of a multi-layer material (MLM) with the properties of materials from which individual layers are made.

#### 2.1.2. Post-Consumer PVC Recycling

Another issue is the management of post-consumer waste. Excellent resistance to weather conditions and the process of aging make PVC products, such as window profiles, construction profiles, and pipes and cable insulations very long-lasting. An increased supply of waste from these products could be expected in the near future, as their 30–40 years of use are coming to an end. Even though the PVC in this waste is not significantly degraded and could constitute a valuable raw material for recycling, there may be obstacles in their management. Over the years of using PVC products, legal regulations have changed that prohibit the use of certain chemical compounds, such as additives to polymers. In the case of PVC, lead-based (Pb) stabilisers and some phthalate plasticisers are particularly problematic [41,42,43]. Solutions are being developed to effectively separate these compounds from PVC recyclates.

Products with a short lifetime (less than 2 years) constitute only 15% of the total amount of PVC products [44]. These are mainly bottles and containers. PVC is also used to produce labels for packaging made of other polymer materials, in particular PET beverage bottles, packaging for drugstore and household chemicals made of PP and PE. The mechanical separation of PVC from such a waste stream is not problematic. Sedimentation and gravimetric methods are excellent for the separation from polyolefins, due to the large difference in density between the materials [45,46,47,48]. In separation from PET, high efficiency is achieved using electrostatic-, flotation- or hardness-differencing methods [49,50,51,52,53,54,55]. For pulverised materials, hydrocyclones can be used [56]. PVC raw material obtained from the recovery process can be successfully processed into a number of new products, in particular polymer composites [57,58,59,60,61].

Wire insulation obtained from waste electronic equipment, household appliances and cars is the source of plasticised PVC recyclate. The mechanical separation of the insulation from the metal core is not a problem [62,63,64,65,66,67,68,69]. A polymer mixture is obtained with PVC as the main polymer [66,70]. It is easy to separate with the already mentioned methods. Due to the lower melting temperature of plasticised PVC compared to other polymers, melt filtration can be successfully used to remove polymer impurities with higher melting temperatures [71,72,73]. Recycled cable insulation materials are difficult to recycle into insulation due to the technical requirements for these materials. The material can be processed into other technical products, including composites with recycled fillers [74,75,76]. Shredded cable insulation is also an additive to cement and bituminous masses [77,78,79,80,81,82,83].

A relatively high amount of plasticised PVC is used in medicine. This is mainly in disposable products. Some of them are considered hazardous materials and need to be incinerated, but many of them are valuable materials that can be reused [84,85].

### 2.2. Feedstock Recycling

**Figure 6:**
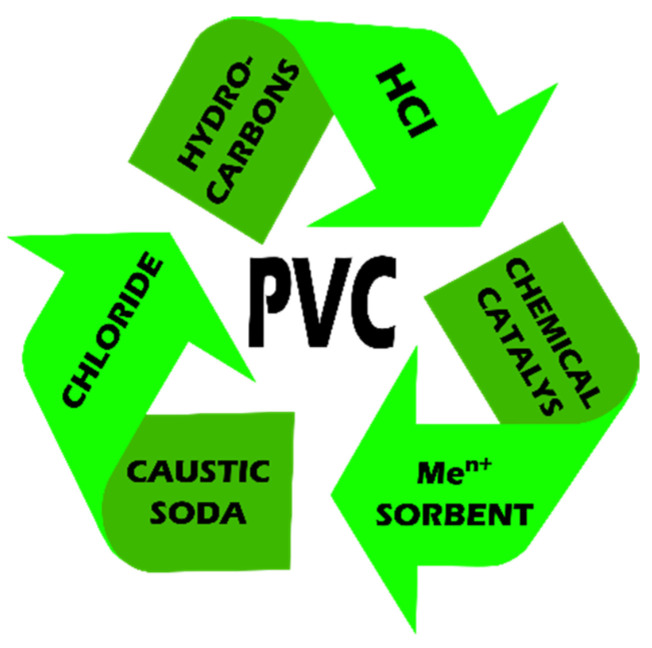
Main directions of substance recovery as a result of feedstock recycling of PVC.

Feedstock recycling is an alternative to mechanical recycling and the disposal of post-consumer waste. It is more suitable for an unsorted PVC waste stream for which material recycling is not achievable or is uneconomical. Its main purpose is to reintroduce raw materials into a closed circuit and recover the energy contained in the material. The chemical substances produced in the process of PVC decomposition have various applications (Figure 6), especially in the chlorine industry [30].

The thermal recycling of PVC waste includes the thermal treatment of the waste stream towards the recovery of hydrogen chloride, which is recycled for the production of PVC or other processes. PVC is a material whose thermal recycling method was indicated as ineffective and therefore not future proof. However, there is currently a lot of intense work aimed at subjecting this waste to thermal recycling. Several thermal recycling processes are used, for example pyrolysis, gasification, incineration and modifications thereof. Many problems in thermal recycling are caused by process additives, such as stabilisers and plasticisers commonly used in PVC processing, which are currently on the list of prohibited substances [86].

Incorrect thermal utilisation of Cl-containing waste, including PVC, may cause significant damage to installations due to the corrosive properties of the resulting gaseous products. The formation of dioxins at unsuitable temperatures is also dangerous, which is why the control of the process is so important.

The thermal treatment of PVC waste essentially consists of two steps: dechlorination to remove Cl from the PVC macromolecule and the use of the remaining hydrocarbons portion. For thermal recycling, dechlorination is necessary to reduce the potential environmental hazards and to increase the recovery of hydrocarbons from PVC waste. Additionally, the neutralisation of HCl in the tail gas is required. Currently, the work on the thermal recycling of PVC is focused on obtaining chlorine, hydrogen chloride and salt. These products are not treated as a waste material causing technical complications but as a full-value source of raw materials for further processes [30]. The issue of chlorine removal from PVC waste before its proper disposal is one of the main research topics of the thermal recycling of waste materials [30,87,88,89,90,91,92,93,94,95,96].

The dechlorination and recovery of Cl during the thermal recycling of PVC, for example, can be completed with ethylene glycol and NaOH [97,98,99]. The resulting NaCl salt and glycol are separated by electrodialysis and reused in various processes. The obtained hydrocarbon fraction can be utilised in thermal treatment or used for further processes, e.g., fuel production. Such a procedure ensures protection against corrosion of the installation and the best energy recovery from the remaining hydrocarbon portion [87,98,100,101,102,103].

Hydrothermal dechlorination with moist biomass is another method of thermally recycling PVC waste [93,100,104,105,106,107]. In the face of an increasingly serious environmental and energy crisis, it has aroused great interest in recent years. The presence of PVC in the process of hydrothermal carbonisation promotes the formation of a higher content of carbon residue [104,105,106,107,108,109,110,111,112], thus increasing the carbonisation of cellulose and coke yield, while reducing the emission of gases and oily substances [104]. PVC biomass co-pyrolysis can also be used to produce sorption materials [93,113], such as chlorinated carbon black used for mercury absorption [114,115], hydrocarbon for methylene blue adsorption in an aqueous medium [107], and porous carbon spheres with high CO_2_ greenhouse gas absorption potential [116].

Waste PVC, due to the reactive chlorine built into the polymer chain, may turn out to be a valuable raw material for the production of efficient sorbents and dangerous, as well as valuable, metal ions [88,117].

There are reports on the catalytic acceleration of the PVC waste dechlorination in the presence of various substances [114,118,119,120,121,122]. The process of dechlorination, by binding chlorine and HCl, is also influenced by additional substances, such as Na_2_CO_3_, KOH, NaOH, NH_3_·H_2_O, CaO and NaHCO_3_ [87,100,101,123,124,125,126,127,128].

Studies are also conducted on the thermal recycling of PVC waste on an industrial scale. They are run by companies such as Solvay, Suez and Resolset. The process uses the technology of chlorine neutralisation (through a dry scrubber with sodium bicarbonate), as a result of which NaCl is obtained, which, after cleaning, is used by Solvay for the production of caustic soda.

Another example of the thermal recycling of PVC waste on an industrial scale are the processes implemented under the Thermo Vinyl project in Switzerland, based on the recovery of energy and HCl by wet scrubbing of the gases formed in the process of the incineration of municipal waste. Hydrochloric acid is reused to extract the metals contained in the ashes after combustion. This process uses the already available infrastructure of waste-treatment plants.

## 3. Summary

**Figure 7:**
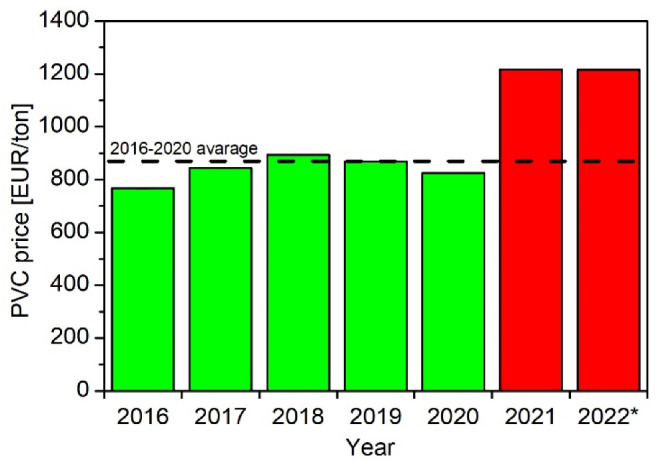
PVC forward-purchase prices on the stock exchange (DPVc1 indexed on Dalian Commodity Exchange) * from January to June.

Figure 7 shows average PVC forward-purchase prices on the stock exchange (DPVc1 indexed on Dalian Commodity Exchange) after converting into EUR/ton at a rate on the listing date [129,130]. The PVC price in the years 2016–2020 was stable, and the average for that period amounted to EUR840 per ton. However, the market recorded a sudden increase in the price of this raw material in 2021. The average price for 2021 and 2022 (January–June) was already EUR1216 per ton, which is a 45% increase relative to the previous 5-year period.

In addition, a continuously growing demand for poly(vinyl chloride) products is observed, primarily in the construction and medical industries. As a consequence, it is economically justified to undertake investments and organisational actions aimed at increasing the material recycling level. Mechanical recycling of manufacturing waste seems particularly well-grounded. It is definitely the easiest, since it concerns a material of a defined composition and properties, and an appropriate manufacturing organisation at a company enables, in many cases, uses already-owned equipment (extruders, mills, agitators).

The advantage of PVC, which is its simple modification, can constitute a significant hindrance in respect of post-consumer waste-material recycling. Developing adequate technologies aimed at separating PVC materials, sometimes exhibiting extremely different properties and compositions or the implementation of their simultaneous processing techniques, and the production of materials of assumed properties are a challenge in this case. When it comes to post-consumer waste, organising waste collection that guarantees raw material availability and quality is a strategic task.

Another challenge is for the PVC waste to include materials produced 30 or even 50 years ago. They can contain already-forbidden process additives, such as thermal stabilisers based on lead compounds and certain plasticisers.

PVC waste that constitutes a problem for material recycling should be subjected to feedstock recycling. However, it requires large investment outlays associated with the need to design and construct adequate industrial systems. Still, in the pursuit of circular economy, even if the profitability of such recycling is very low, investments may be justified for ecological reasons, and the research on their development may also bring tangible financial gains in the future.

The issue of recycling is not indifferent to companies related to the PVC industry. As part of a voluntary VinylPlus initiative, they committed to developing more eco-friendly manufacturing, application and recycling methods, with their activities covering all sectors of the PVC industry. Their actions focus on minimising the impact of production on the environment, promoting the responsible use of process additives, supporting operations related to the continuous development of PVC waste-collection and recycling systems, and progressing towards carbon neutrality. In 2004, the amount of PVC recycled pursuant to the commitments was 18 thousand tonnes. Owing to the joint initiative, as much as 264 thousand tonnes of PVC was recycled in 2010 and 731 thousand tonnes in 2020. It is assumed that, by 2030, the recycling volume would reach 1 million tonnes [131].

Despite the unfavourable opinion, PVC is a material that it certainly recyclable. Furthermore, its recycling level grows year after year. The current PVC-material recycling possibilities and feedstock recycling development perspectives, which would enable processing such waste in the future with a positive environmental and financial effect, do not constitute grounds to exclude the application of poly(vinyl) chloride in the era of sustainable development and the desire to create circular economy.
